# Reply to Kelman: The foundations for studying catastrophic climate risks

**DOI:** 10.1073/pnas.2214794119

**Published:** 2022-10-10

**Authors:** Luke Kemp, Chi Xu, Joanna Depledge, Kristie L. Ebi, Goodwin Gibbins, Timothy A. Kohler, Johan Rockström, Marten Scheffer, Hans Joachim Schellnhuber, Will Steffen, Timothy M. Lenton

**Affiliations:** ^a^Centre for the Study of Existential Risk, University of Cambridge, Cambridge CB2 1SB, United Kingdom;; ^b^Darwin College, University of Cambridge, Cambridge CB3 9EU, United Kingdom;; ^c^School of Life Sciences, Nanjing University, Nanjing 210023, China;; ^d^Cambridge Centre for Environment, Energy and Natural Resource Governance, University of Cambridge, Cambridge CB2 3QZ, United Kingdom;; ^e^Center for Health and the Global Environment, Hans Rosling Center for Population Health, University of Washington, Seattle, WA 98195;; ^f^Future of Humanity Institute, University of Oxford, Oxford OX2 0DJ, United Kingdom;; ^g^Department of Anthropology, Washington State University, Pullman, WA 99164;; ^h^Santa Fe Institute, Santa Fe, NM 87501;; ^i^Cluster of Excellence ROOTS, Christian-Albrechts-Universität, 24118 Kiel, Germany;; ^j^Potsdam Institute for Climate Impact Research, 14473 Potsdam, Germany;; ^k^Department of Environmental Sciences, University of Wageningen, 6708PB Wageningen, The Netherlands;; ^l^Earth System Science Department, Tsinghua University, Beijing 100190, China;; ^m^Fenner School of Environment and Society, The Australian National University, Canberra, ACT 2601, Australia;; ^n^Global Systems Institute, University of Exeter, Exeter EX4 4QE, United Kingdom

We appreciate Dr. Kelman’s contribution “Connecting Disciplines and Decades”’ (1) in response to “Climate Endgame”’ (2). We naturally agree with Dr. Kelman that exploring catastrophic climate scenarios is vital, neglected, and possible.

We further agree that exploring catastrophic climate scenarios requires interdisciplinary work informed by existing research. There is a rich history to draw on when studying catastrophic climate risks. Kelman highlights some of these, but there are deeper and broader roots. In sociology, there is not only Perrow’s normal accidents theory but also the concept of the risk society (3). In statistics, the pertinent area of extreme value theory underwent intensive development in the 1920s and can trace a longer lineage to the 18th century (4). Historical exploration of societal collapse and transformation dates to at least the 18th century as well (5), with a blossoming literature after the 1980s. While many of these ideas are relevant to the study of extreme climate risks, few look at the outcome of human extinction. Attention to this emerged namely post-1954 in the wake of the Castle Bravo nuclear test (6). Systematic scholarly work largely began in the 1990s with John Leslie’s 1996 *The End of the World* (7).

“Climate Endgame” is a perspectives piece, aiming to articulate a rationale and an approach for the intensive study of catastrophic climate risk. We did not attempt to comprehensively catalog all the relevant literature or to exhaustively trace the history of mentions of extreme climate risks. Instead, we call for future efforts to do so, for example as part of the proposed Intergovernmental Panel on Climate Change special report. We have, we hope, identified highly relevant studies and provided a rationale, lexicon, and sample approach for studying these scenarios, thus providing a foundation for more work in this area.

The ethos expressed in “Climate Endgame” of taking extreme risks seriously, even if they are low probability, is a familiar notion in fields such as disaster risk management. Nonetheless, it is one that is rarely applied in climate risk analysis. Moreover, it is rarely extended to the most catastrophic outcomes that we highlight, such as human extinction and collapse. This leaves an important gap.

We would like to underscore Kelman’s point that understanding extreme risks means engaging with value pluralism, recognizing that risks are informed by societal values (8). This is why we call for the use of deliberative democratic methods to evaluate responses to extreme risks. Such approaches are vital to tackling catastrophic risks. They can help accommodate value pluralism, inclusively define risk, and ensure democratic safeguards (9). Drawing on historical perspectives and ensuring cultural diversity can further strengthen such approaches.

Finally, we would like to take issue with the claim that the application of concepts such as planetary boundaries and tipping points in our paper is “uncritical.” These have robust bodies of underpinning literature, including responses to the criticism that Kelman cites (10). Crossing tipping points and planetary boundaries is high-risk and potentially irreversible (11). Like extreme risk analysis, these require serious consideration rather than dismissal.
